# PROTOCOL: Effects of social prescribing for older adults: An evidence and gap map

**DOI:** 10.1002/cl2.1382

**Published:** 2024-02-29

**Authors:** Elizabeth Tanjong Ghogomu, Vivian Welch, Mojde Yaqubi, Omar Dewidar, Victoria I. Barbeau, Srija Biswas, Kiffer Card, Sonia Hsiung, Caitlin Muhl, Michelle Nelson, Douglas M. Salzwedel, Marianne Saragosa, Cindy Yu, Kate Mulligan, Paul Hébert

**Affiliations:** ^1^ Bruyère Research Institute University of Ottawa Ottawa Canada; ^2^ Bruyère Research Institute Ottawa Canada; ^3^ Canadian Institute of Social Prescribing Canadian Red Cross Toronto Canada; ^4^ Faculty of Health Sciences Simon Fraser University Vancouver Canada; ^5^ School of Nursing, Faculty of Health Sciences Queens University Kingston Canada; ^6^ Dalla Lana School of Public Health University of Toronto Toronto Canada; ^7^ Department of Anesthesiology, Pharmacology and Therapeutics University of British Columbia Vancouver Canada; ^8^ Lunenfeld‐Tanenbaum Research Institute Sinai Health Toronto Canada; ^9^ GenWell Project Ottawa Canada; ^10^ Centre Hospitalier de l'Université de Montréal Montreal Canada

## Abstract

Objectives

This is the protocol for an evidence and gap map. The objectives are as follows: The aim of this evidence and gap map is to map the available evidence on the effectiveness of social prescribing interventions addressing a non‐medical, health‐related social need for older adults in any setting.

Specific objectives are as follows:

1. To identify existing evidence from primary studies and systematic reviews on the effects of community‐based interventions that address non‐medical, health‐related social needs of older adults to improve their health and wellbeing.
2. To identify research evidence gaps for new high‐quality primary studies and systematic reviews.
3. To highlight evidence of health equity considerations from included primary studies and systematic reviews.

## 1 BACKGROUND

### 1.1 Introduction

#### 1.1.1 The problem, condition or issue

With increasing life expectancy, the global population is expected to reach 2 billion people over the age of 60 years by 2050 (WHO, 2020). Low socioeconomic status, declining physical, function, and cognitive capacity occur with age resulting in complex functional, social and health needs, and multimorbidity for many older adults, increasing the demand for health and social services (Elston, 2019; Weaver, 2020; WHO, 2015) to improve their wellbeing and functional ability (Elston, 2019; WHO, 2015). Non‐medical factors or social determinants of health including socioeconomic status, unemployment, education, social support, social exclusion, and transport affect health outcomes and wellbeing of older adults (Andermann, 2016; Elston, 2019; Weaver, 2020). Addressing these factors may lead to healthy ageing which is the process of developing and maintaining the functional ability that enables wellbeing in older adults (WHO, 2015) even those who have chronic conditions.

General practice is a central access point of health systems and consultation patterns have shown persistent frequent attendance of older adults with medical and social problems (Welzel, 2017) which affect health, defined as the state of complete physical, mental and social wellbeing and not merely the absence of disease or infirmity (WHO, 2002). Over 20% of visits, according to general practitioners in the UK, are spent addressing patients’ psychosocial issues such as financial, housing, employment, or social relationship and support network problems (Elston, 2019; Kiely, 2022; Zantinge, 2005). Challenges arising from a disadvantaged social environment and lifestyle may result in negative psychological, physical, and social outcomes such as depression or loneliness (Brandling, 2009). Medical treatments for depression and other health issues may be pragmatic, but they can easily become a very demanding option for health systems when the underlying psychosocial problems are not addressed. General practioners may not routinely address these issues due to consultation time constraints or a lack of knowledge of available resources and partnerships with other sectors that can address them (Andermann 2016; Pescheny, 2020).

One of the interventions introduced to help health professionals manage these problems is social prescribing. The term originated in the UK and is being adopted internationally in high‐income countries (Australia, Canada, Germany, Japan, Netherlands, Portugal, Singapore, Spain, USA) especially but also in some middle‐income countries such as China, India, Iran, and Malaysia (Khan, 2023; Morse, 2022). It describes interventions that connect individuals to non‐clinical services and activities typically offered by voluntary and community sectors to improve health and wellbeing (Khan, 2023; Morse, 2022; WHO, 2022). Similar interventions have been developed in other countries, but not not called social prescribing (Morse, 2022), for example, care navigation (Tierney, 2019) and community health programs (Khan, 2023). There is no commonly accepted definition for social prescribing, and the activities or support services that constitute social prescribing are sometimes recommended as social prescriptions (Khan, 2023; Morton, 2015; Rempel, 2017; Vogelpoel, 2014) and vary depending on the person's needs and the community and care settings. However, a recent Delphi study, through a global consensus, has established a definition for social prescribing as a means for trusted individuals in clinical and community settings to identify that a person has non‐medical, health‐related social needs and to subsequently connect them to non‐clinical supports and services within the community by co‐producing a social prescription—a non‐medical prescription, to improve health and wellbeing and to strengthen community connections (Muhl, 2023). Models of social prescribing rely on the principles of health promotion and addressing social determinants of health, such as socioeconomic status, social inclusion, housing, and education, to improve health outcomes by connecting patients with non‐clincal sources of support (Morse, 2022; Mulligan, 2020; WHO, 2022).

Given that only 10%–20% of the modifiable contributions to population health outcomes are thought to be accounted for by medical care (Hood, 2016), several national and international policy organizations such as the National Health Service (NHS) England, National Institute of Health Research UK (Tierney, 2020), and the World Health Organization (WHO) (WHO, 2022) advocate for social prescribing as an important avenue for expanding the treatment options. Social prescribing has been proposed as an option for available general practitioners and other community‐based practitioners to address psychosocial factors and refocus the healthcare system by moving people away from the medicalization of social needs (Adbowale, 2014; WHO, 2022).

Social prescribing can target anyone but populations with needs rooted in the social and structural determinants of health (such as income security, housing, belonging, or discrimination), and intersecting health equity factors (such as place of residence, race, ethnicity, class, gender, sexuality, disability, or age) benefit most (Fixsen, 2022). These populations include older adults who are at risk of social isolation or experience loneliness due to health and social inequality issues such as chronic health conditions, disability, socioeconomic deprivation, digital exclusion, or belonging to marginalized groups (e.g., racialized or ethnic minority groups, LGBTQ2S+) (WHO, 2022). Potential benefits of social prescribing for ageing population and the health system include improving their health and wellbeing, reducing their social isolation and loneliness, contributing to their resilience, increasing their wellbeing, delaying/preventing admission to hospital, and improving systems (Hamilton‐West, 2020). However, they are often hard to reach and tend to be under‐represented in research (Forsat, 2020).

Social prescribing provides a model of health that can mitigate the effects of unmet social needs and address health inequalities (Chatterjee, 2018). Addressing social needs has gained greater attention given how the COVID‐19 pandemic has placed people, especially older adults, at increased risk of social isolation, with physical and mental health impacts, due to public health policies limiting their access to community services and family support (Haines, 2020; Hwang, 2020). Before the COVID‐19 pandemic, social prescribing was increasingly provided to older adults given their increased likelihood of experiencing depression, increased cognitive impairment, or mobility impairment in the presence of elevated social risk (Del, 2020; Del, 2022; O'Toole, 2016). The pandemic has prompted the consideration of social prescribing on national and international levels and demonstrated the value of strong community social support in addressing public health concerns of vulnerable populations, including older adults (Younan, 2020).

#### 1.1.2 The intervention

Social prescribing usually involves screening for non‐medical, health‐related social needs and connecting to non‐clinical supports and services that are typically provided by community‐based organizations to improve health and wellbeing, and to strengthen community connections (Muhl, 2023). It may be operationalised differently across the globe. For instance, the identification of non‐medical, health‐related social needs is not always by a health care provider in clinical settings; it could be conducted by a trusted individual in the community setting. The identifier could do the following: (1) directly connect the person to community supports and services; (2) or refer the person to a connector who connects them to non‐clinical community supports and services.

Other terms used in the literature for the connector include link worker, well‐being coordinator, community connector, community health worker, patient or care navigator, social prescriber, health facilitator, or well‐being coach (Hamilton‐West, 2020; Morse, 2022). Tierney et al. found 75 different terms used in the literature (Tierney, 2019).

Social prescribing creates a trackable pathway for identifiers and connectors to connect people to community‐based support and services and follow them up (Mulligan, 2020). Although signposting, which is the provision of a list of resources, is considered on the light touch side of the intensiveness spectrum (Howarth, 2019; Kimberlee, 2015), it involves people contacting the services they need or self‐referral, which might be challenging for some (White, 2022). Therefore, social prescribing is recently thought to be more than signposting and distinct (Husk, 2020; Morse, 2022; White, 2022), although both terms are sometimes used interchangeably (Morse, 2022). Care navigation is another term used interchangeably with social prescribing. Care navigation is defined as an avenue to link patients to activities or organizations that can help address non‐medical needs affecting health and wellbeing (Tierney, 2019). It originated in the USA and Canada to help patients with complex needs or socially disadvantaged groups to access health services (Brunton, 2022; Carter, 2018) through signposting (Brunton, 2022; Tierney, 2019). Like social prescribing, care navigation connects people to community‐based resources, improves health and wellbeing and reduces healthcare demand (Brunton, 2022).

The community‐based resources vary depending on the needs of the patients. Examples of services include art therapy, walking and exercise classes, nature‐based activities, volunteering, as well as employment, debt, housing, and legal advice (Bickerdike, 2017; Kimberlee, 2013; Morse, 2022). Different models for social prescribing, or a continuum, have been described in the literature (Husk, 2020; Kimberlee, 2015). For example, social prescribing could encompass signposting, light, medium, or holistic models (Howarth, 2019; Kimberlee, 2015).

- Social prescribing as signposting, where a person is advised to participate in a community activity (e.g., knitting group) or provided with a list of resources in the community that they can choose to access and follow through.
- Social prescribing light: at‐risk or vulnerable persons are referred to specific programmes to address a specific need or to attain a specific objective (e.g., exercise on prescription).
- Social prescribing medium involves a single or small number of conversations with a connector to understand what matters to the person, provide advice, and determine an appropriate referral to address the person's health‐related social needs.
- Social prescribing holistic involves partnership work between the identifier, the connector, and the person. The identifier refers the person to the connector who has unlimited conversations with the person, understands what matters, co‐produces a personalized action plan, supports the person to access community supports and services, follows up with the person and reports back to the identifier.

The holistic model is person‐centred and based on integrated care with a multidisciplinary team working in collaboration with voluntary and community sectors (Howarth, 2019; Morse, 2022). These sectors provide complex interventions that recognize the intersection of social determinants of health while using various activity types such as income, employment, education, housing, and social support that are beyond the circle of medical care. The interventions address all the needs that the person has to improve their health and wellbeing.

The impact of social prescribing varies depending on the delivery scheme (Pescheny, 2020). For example, White et al. report on the effects of referring patients with severe mental health issues on health outcomes and found limited improvements to patients’ well‐being (White, 2010). The intervention scheme was made to help people solve problems themselves rather than getting the problem solved. Alternatively, other randomized controlled trials (RCTs) and observational studies have demonstrated positive effects of social prescribing on patient's health and well‐being (Jensen, 2018; Maughan, 2016) and reduced healthcare use (Carnes, 2017; Loftus, 2017). Jensen & Bond assessed the effects of arts and creative activities while Maughan et al., assessed the impact of various services including self‐management resources, educational, leisure and recreational facilities and fitness‐, health‐ and exercise‐related activities in conjunction with raising awareness of mental health. Carnes et al. assessed the effects of a social prescribing service involving developing an action plan and assigning a volunteer if necessary to help patients achieve their goals. They found reduced General Practitioner consultation rates after 1 year in the intervention group, although there were no differences in general health, depression, anxiety and ‘positive and active engagement in life’ between the intervention and control groups. Loftus et al. found that participation in social prescribing activities including social clubs, men's shed, counselling, arts program, craft classes, crochet classes, falls prevention, exercises, befriending, personal development, and computer courses reduced healthcare use compared to controls.

#### 1.1.3 Why it is important to develop the EGM

Many of the evaluations of social prescribing schemes are small‐scale with poor methodologies and show little evidence of their effectiveness (Bickerdike, 2017; Hamilton‐West, 2020; Kiely, 2022; Pescheny, 2020; Woodhall, 2018). It is difficult to evaluate social prescribing due to the complex nature of the interventions. Even though several studies have been conducted on social prescribing, researchers have drawn attention to the methodological and generalizability limitations of the available evidence for older adults (Hamilton‐West, 2020). The literature on social prescribing is sparse and based on operational elements. Programs for connecting people with non‐clinical community services are not always called social prescribing and may sometimes be labelled as community connector programs, community referral, community links, social referral, or social prescription (Rempel, 2017; Thomson, 2015). Furthermore, older adults may have unique preferences or challenges for social prescribing to support connection back to the community.

Emerging work on social prescribing shows global promise as a low‐cost, scalable health promotion intervention that links health and social systems; tracks the impacts of social interventions on the wellbeing of participants, staff and volunteers; promotes health and reduces systems costs by moving care upstream; and improves health equity by supporting the capacity of people and communities to make choices and exert more control over the context‐specific circumstances of their own health and wellbeing (Carnes, 2017; Dowden, 2019; Morse, 2022).

Despite rapidly growing interest and several local and regional social prescribing initiatives, social prescribing has not yet emerged as a systems‐level intervention in most countries (Morse, 2022). For example, different models exist across Canada and the recently created Canadian Institute for Social Prescribing (CISP) is in the process of developing a national framework for social prescribing in Canada (Khan, 2023). There is an immediate need to understand the practical context; map existing and emerging social prescribing initiatives; define common languages, practices, and indicators; link to global scale social prescribing research and policy frameworks; and ensure the full incorporation of health equity and community engagement throughout the growth and development of social prescribing models (Hamilton‐West, 2020; Woodhall, 2018).

The COVID‐19 pandemic has demonstrated an immediate need for new and better‐connected interventions that identify, and address needs rooted in the social determinants of health, including individual social connectedness and collective social cohesion (Ndumbe‐Eyoh, 2021). Social determinants of health are especially crucial for older adults who are classified as a high‐risk group for experiencing loneliness with negative effects on mental health and wellbeing (Kim, 2021). As the world moves into the next stages of the pandemic, there is a need to remodel social prescribing to improve social connections and community resilience by encouraging mutual support and improving access to community services (Younan, 2020). There is also a need to build collective capacity that supports the sustainability and wellbeing of a health and social services workforce experiencing burnout and moral distress (Spilg, 2022; Sriharan, 2021; Sukhera, 2021).

Given that social prescribing is still novel with a recent global Delphi definition and an emerging evidence base, we will include all approaches that meet the global Delphi definition for social prescribing even if they are not called social prescribing. The map will examine up to date evidence of effectiveness from systematic reviews as well as primary studies to identify gaps and clusters in interventions and outcomes assessed. It will improve the discoverability of available evidence by stakeholders including health and social care providers, the public, policymakers, and relevant organizations such as the Canadian Institute of Social Prescribing (CISP). This map will inform prioritization of future research for areas where there are gaps. It will also inform future evaluations and guide decisions about investing in social prescribing.

#### 1.1.4 Existing EGMs and/or relevant systematic reviews

To our knowledge, there is only one evidence gap map on social prescribing assessing improvements in health, wellbeing, and healthcare use in patients of all ages (Price, 2017). The evidence and gap map covered studies published up to December 2016 and did not focus on older adults. The gap map also included signposting programs to deliver social prescribing.

Several reviews have assessed the effects of social prescribing (Bickerdike, 2017; Chatterjee, 2018; Cooper, 2022; Napierala, 2022; Percival, 2022; Smith, 2019; Zhang, 2021) with mixed findings and only two were focused on older adults. In 2019, Smith et al. assessed the impact of social prescribing on preventing or delaying frailty in community‐dwelling adults and did not find any eligible reports. However, a recent review led by Percival et al. (2022) assessing social prescribing on older adults in any setting identified only seven studies with considerable positive effects on physical and psychosocial outcomes, depending on the health resource use, although all studies had a considerable risk of bias. These studies assessed interventions labelled as social prescribing.

We aim to develop a more comprehensive and up to date map that will include social prescribing, social prescription, signposting and care navigation activities related to social prescribing and focused on older adults.

## 2 OBJECTIVES

The aim of this evidence and gap map is to map the available evidence on the effectiveness of social prescribing interventions addressing a non‐medical, health‐related social need for older adults in any setting.

Specific objectives are as follows:

1. To identify existing evidence from primary studies and systematic reviews on the effects of community‐based interventions that address non‐medical, health‐related social needs of older adults to improve their health and wellbeing.
2. To identify research evidence gaps for new high‐quality primary studies and systematic reviews.
3. To highlight evidence of health equity considerations from included primary studies and systematic reviews.

## 3 METHODS

We will adhere to the Campbell Collaboration guidance for producing evidence and gap maps (White, 2020).

### 3.1 Evidence and gap map: Definition and purpose

Evidence and gap maps are a type of systematic evidence synthesis product that visually present the existing evidence and its quality relevant to a specific research question (Snilstveit, 2013; White, 2020).

The evidence and gap map typically presents findings in a two‐dimensional matrix with interventions as row headings and outcomes as column headings (Snilstveit, 2016; White, 2020). Each cell shows the studies with evidence corresponding to the intervention and outcome of interest. This map will also highlight areas where evidence is lacking for the effects of social prescribing on older adults.

### 3.2 Framework development and scope

The development of the intervention‐outcome framework for this EGM was informed by existing strategy documents and research studies, and consultation with key stakeholders.

We identified and reviewed existing frameworks and typology of intervention and outcome categories used in the following documents: the WHO toolkit on how to implement social prescribing (WHO, 2022), the social prescribing pathway by Bridgeable (Bridgeable, 2022), the Common Understanding of Social Prescribing (CUSP) framework (Muhl, 2023), National Academy for Social Prescribing resources, the evidence and gap map on social prescribing by Price (Price, 2017), and some research studies about social prescribing (Chatterjee, 2018; Hamilton‐West 2020; Polley, 2020).

Since social prescribing is a means to connect patients to a range of non‐clinical supports and services in the community to improve health and wellbeing, we defined the intervention categories by the types of non‐clinical supports and services provided to meet the needs of older adults. The five main categories include lifestyle support, psychosocial support, material support, arts based activities, and nature. We will also consider others including population specific or culturally safe interventions, signposting programs and care navigation.

We considered process outcomes, individual level outcomes related to health and psychosocial wellbeing, health system usage and cost, as well as community level outcomes and adverse effects.

The scope of this EGM is to cover all evidence on the effectiveness of social prescribing interventions in older adults in any setting.

### 3.3 Stakeholder engagement

We consulted a Canadian stakeholder advisory group to define the scope and develop the framework for the map. The members were academics, researchers, decision makers, healthcare professionals and providers of social prescribing from key organizations including Abilities Center, the Canadian Coalition for Seniors Mental Health (CCSMH), the Canadian Institute of Social Prescribing (CISP), Canadian Red Cross, Healthy Aging Alberta, United Way British Columbia, United Way Centraide Canada, Victorian Order of Nurses (VON), as well as University of Alberta, University of Toronto, and Queen's University.

These individuals will be consulted throughout the development of the evidence and gap map to provide input on the revised framework, preliminary findings, and draft map. We will also share preliminary findings and the draft map with other stakeholders and users of social prescribing services and programs beyond Canada including the National Academy of Social Prescribing (NASP) and the Global Initiative to End Loneliness, to seek their feedback.

### 3.4 Conceptual framework

Older adults are susceptible to unmet needs due to functional impairment, declining physical and cognitive health, and chronic illnesses (Abdi, 2019; Hamilton‐West, 2020). These needs require care and support that can be provided through a holistic approach addressing complex physical and psychological health and social challenges that older adults face. While there are community support services and programs, they are not readily available and accessible to older adults (Hamilton‐West, 2020) leading to increased healthcare utilization and costs (Clements‐Cortés, 2020). Social prescribing is one of the strategies that complements health care by increasing access to non‐clinical community support services and programs to help older adults improve their health and wellbeing, to reduce the burden on the healthcare system (Clements‐Cortés, 2020; Elston, 2019; Hamilton‐West, 2020; Muhl, 2023; Percival, 2022) and to strengthen community connections (Muhl, 2023).

**Figure 1:**
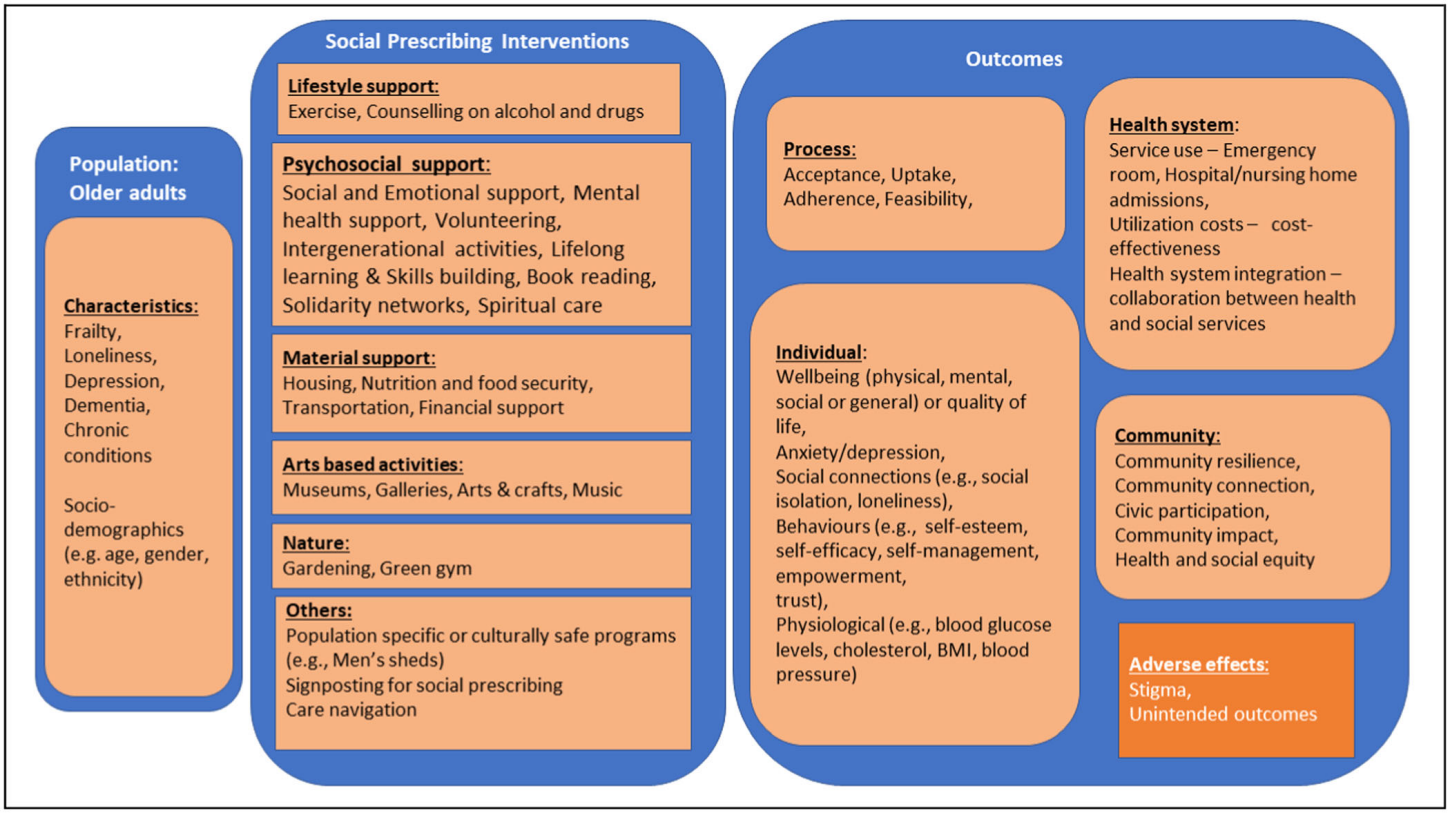
Conceptual framework.

The conceptual framework for this EGM (Figure 1) is a simplified diagram describing how social prescribing can meet the complex needs of older adults living with chronic conditions to achieve process outcomes as intermediate outcomes (e.g., acceptance, feasibility, uptake, adherence) and longer‐term outcomes including individual‐level outcomes (e.g., improved health and wellbeing), health system‐level outcomes (e.g., utilization and cost), and community‐level outcomes (e.g., community connection, civic participation, resilience) by turning motivation into action.

Healthy ageing and wellbeing are determined by health conditions such as frailty, depression, dementia, chronic diseases, and sociodemographic characteristics like age, education, sex, socio‐economic status, as well as physical and social environments (Clements‐Cortés 2020; WHO, 2015). By assessing and understanding the state of wellbeing and needs of older adults, an identifier (health practitioners or community workers or volunteers/trusted individuals in the community) can refer them to appropriate community services and programs which can improve their health and wellbeing or refer them to a connector (community link worker or navigator) who will support them to identify their needs and appropriate services to engage with (Elston, 2019; Muhl, 2023; Percival, 2022).

Several models of social prescribing exist, and the theoretical underpinnings have been described using the self‐determination theory of motivation (Bhatti, 2021; Hanlon, 2021; Morse, 2022). The motivation to maintain health and wellbeing is influenced by social and environmental factors (Morse, 2022; Ryan, 2000). The acceptance, uptake, and adherence to services and programs to improve wellbeing are linked with self‐determination or self‐motivation and depend on the satisfaction of basic psychological needs including autonomy (the need to feel control over one's activities and behaviours, or life and decisions), competence (the ability to influence outcomes, be capable, and effective), belonging/relatedness (the need to have close, affectionate relationships), and beneficence (having a positive impact on others) (Bhatti, 2021; Hanlon, 2021; Husk, 2020; Morse, 2022). Other factors may influence uptake and adherence to programs and behaviours such as availability and visibility of resources, social norms, cultural expectations, education, health literacy, physical, material, or structural barriers to change (Bhatti, 2021; Morse, 2022; Pescheny, 2018). In addition, building a trusted relationship between the identifier/connector and the individual may facilitate the implementation of social prescribing and improve acceptance and uptake (Bhatti, 2021; Pescheny, 2018; White, 2022). However, identifiers and connectors must be skilled to reduce the risk of dependency while establishing trust and build self‐esteem and empowerment in their clients (Bhatti, 2021; White, 2022).

Social prescribing interventions include the following categories of community services and programs:

- Lifestyle support: for example, exercise on prescription, counselling for alcohol and drugs (Bickerdike, 2017).
- Psychosocial support: for example, social and emotional support, volunteering, intergenerational activities, lifelong learning and skills building, solidarity networks or support groups, spiritual support, book reading, storytelling (WHO, 2022).
- Material support: for example, housing, nutrition and food security, transportation, financial support, employment, legal advice and support, citizens advice and support (WHO, 2022).
- Arts‐based activities: for example, music, museums, galleries, arts and crafts, dance, poetry (WHO, 2022).
- Nature: for example, gardening (Kim, 2021), green gym (Thomson, 2015).
- Others:

○ Population specific or culturally safe interventions, for example, men's sheds (Kelly, 2021), Black‐focused social prescribing project in Ontario, Canada (BHC, 2022).
○ Signposting programs to deliver social prescribing (Howarth, 2019; Kimberlee, 2015)
○ Care navigation, for example, case management support for health conditions such as dementia (Challis, 2010), cancer (Ramirez, 2020) or diabetes (Esmene, 2020).

Engaging in social prescribing results in different types of outcomes which include:

- Process outcomes—acceptance, (enrolment), uptake (engagement), adherence, feasibility.
- Individual level—physical/mental/social/general wellbeing or quality of life, anxiety/depression, social connections (social isolation, loneliness), behaviours (self‐esteem, self‐efficacy, self‐management, empowerment, trust), physiological outcomes (blood glucose levels, cholesterol, body mass index (BMI), blood pressure).
- Health systems—service use, service utilization cost, cost‐effectiveness, health system integration, wellbeing of staff.
- Community level—community resilience, community connection, civic participation, community impact, health and social equity.
- Adverse effects—stigma, unintended outcomes.

### 3.5 Dimensions

#### 3.5.1 Types of study design

We will include completed or on‐going systematic reviews and primary studies including RCTs and evaluative quasi‐experimental studies. We will also include systematic and scoping reviews based on their PICO question if they consider effectiveness and explicitly describe adequate search methods used to identify studies, eligibility criteria, methods for critical appraisal and synthesis approaches (Moher, 2015).

We will include quasi‐experimental studies if the participants were allocated through alternate assignment, included threshold on a continuous variable (regression discontinuity) or exogenous variation in the treatment allocation (natural experiments) or other rules including self‐selection by investigators or participants, provided data was collected contemporaneously in a comparison group (non‐equivalent comparison group design), or before and after a discrete intervention (pre‐post analysis design).

We will include eligible studies irrespective of the publication status.

We will exclude primary studies lacking a comparative analysis over time such as cross‐sectional studies. We will exclude qualitative research without a pre‐post evaluation.

#### 3.5.2 Types of intervention/problem

We will consider all types of social prescribing intervention schemes addressing a non‐medical, health‐related need through referral and connection to the community service or program as well as social prescription interventions addressing a non‐medical, health‐related need through connection to the community service or program but there was no referral pathway. The interventions can be conducted in any setting.

We will include interventions which involve or do not involve connectors (Muhl, 2023). Accordingly, we will include studies which involve no connectors, or any trusted individual in clinical and community settings as connectors, or technology‐based interventions delivered to the patients by telephone or digital methods.

We will consider six categories including lifestyle support, psychosocial support, material support, arts‐based activities, nature, and others (population specific or culturally safe interventions, signposting, and care navigation).

We will consider interventions that are provided in the community or clinical setting to address a non‐medical, health‐related need whether they are labelled as social prescribing or not. We will indicate whether the intervention is social prescribing or social prescription. However, in this EGM, we will exclude interventions or programs that are provided strictly from a clinical perspective in clinical settings or community settings as part of routine medical care with no social considerations, for example, substance abuse consultation service for hospitalized patients, routine outpatient palliative care, post‐stroke home‐based rehabilitation by physiotherapists/occupational therapists. See Table 1 for intervention categories and examples.

**Table 1:** Intervention categories.

Intervention categories	Subcategories/examples
Lifestyle support	Exercise
	Counselling for alcohol and drugs
Psychosocial support	Social and emotional support for loneliness/social isolation
	Community‐based mental health support
	Volunteering
	Intergenerational activities
	Lifelong learning (education and skills building)
	Book reading (bibliotherapy)
	Solidarity networks and social support groups
	Spiritual care
Material support	Housing
	Nutrition and food security
	Transportation
	Financial support
	Legal and citizen advice and support
	Employment services, vocational learning, and social entrepreneurship support
Arts‐based activities	Music
	Museums, galleries, arts and crafts
	Dance
Nature	Gardening (horticulture)
	Local parks prescription
	Green gym/Deprescribing for greenhouse gas reductions
Others	Population specific or culturally safe programs (e.g., Men's shed, Black‐focused social prescribing program)
	Signposting for social prescribing
	Care navigation programs (e.g., case management support)

#### 3.5.3 Types of population (as applicable)

We will include studies that focus on older adults, as defined by the WHO—individuals 60 years of age or older (WHO, 2020). Studies including younger and older adults will be included. Studies that do not provide the age of the participants will also be included if they focus on older adults or have no age restrictions (e.g., excluding older adults).

#### 3.5.4 Types of outcome measures (as applicable)

We will not restrict inclusion of studies by outcomes. However, eligible studies and systematic reviews must have a focus on social prescribing. We aim to assess all outcomes related to wellbeing and health such as psychosocial outcomes and behavioural outcomes. All health‐related endpoints such as morbidity, mortality, risk factors, any measures of mental health, as well as any measures of subjective well‐being, quality of life and healthcare utilization and costs will be considered. We will also assess adverse effects associated with social prescribing. See Table 2 for examples of outcome categories.

**Table 2:** Outcome categories.

Outcome categories	Examples	Example of measurements
Process outcomes	Acceptance	Number of people agreeing to the referral or recommendation by a connector; Various survey tools to measure acceptance
	Uptake	Number of people attending an initial appointment with a link worker; Participants’ attendance at activities to which they were subsequently referred or recommended by a connector
	Adherence	Participants’ on‐going attendance
	Feasibility	Various survey tools to measure feasibility
	Wellbeing (physical, mental, social, general)/quality of life	Warwick Edinburgh Mental Well‐being Scale (WEMWBS), Patient Health Questionnaire‐9 (PHQ‐9), EQ. 5D, WHO‐5, SF‐12
	Anxiety/depression	Hospital Anxiety and Depression Scale (HADS); Geriatric Depression Scale (GDS)
	Loneliness	UCLA scale, De Jong Gierveld Loneliness Scale, Campaign to End Loneliness Measure
Individual outcomes	Social isolation	Lubben's Social Network Scale, Social Network Index, PROMIS social isolation 6‐I scale
	Social support	Social support scale; Duke‐UNC functional social support questionnaire
	Social participation	Social Participation Scale
	Physical activity	Self‐reported frequency of physical activity; International Physical Activity Questionnaire (IPAQ)
	Self‐esteem/self‐management	Rosenberg Self‐esteem scale, Self‐efficacy scale, Self‐management assessment scale
	Empowerment	Survey tools to measure positive decision making or activation e.g., Patient activation measure (PAM)
	Trust	Survey tools to measure trusted and supportive relationship with staff
	Physiological outcomes	Blood glucose levels, Cholesterol, BMI, blood pressure
Health system outcomes	Social/Health service use	Number of visits to a General Practitioner (GP), referring health professional, and primary or secondary care services; inpatient admissions; Emergency Room (ER) attendances
	Health/social care utilization costs	Average cost/person; total mean/running costs; environmental costs
	Cost‐effectiveness	Cost‐effectiveness analysis; cost‐benefit analysis, cost‐utility analysis
	Health system integration	Collaboration between health and social services; shifted resources from acute to preventive and social care
	Care team wellbeing/experience	
Community outcomes	Community resilience	Community resilience scale
	Community connectedness	Community belonging; Community Link Evaluation
	Civic participation	Civic engagement scale
	Community impact	Impact on self and others
	Health and social equity	
Adverse effects	Stigma	Number of adverse events
	Unintended outcomes	Number of adverse events

#### 3.5.5 Other eligibility criteria

##### Types of location/situation (as applicable)

We will include all country settings as defined by the World Health Organization regions (Africa, Regions of the Americas, South‐East Region, European Region, Eastern Mediterranean Region, Western Pacific Region) (WHO, 2019) and the World Bank classification by income: low‐income economies, lower‐middle income economies, upper‐middle income economies, high‐income economies (World Bank, 2023).

We will not exclude primary studies and systematic reviews that did not report the country of conduct.

##### Types of settings (as applicable)

We will include studies conducted in all types of community settings such as community centres or parks, art museums, community‐dwelling (residential or personal home), supportive care institutions (long‐term care or nursing home and assisted living facilities) and clinical settings if the interventions address a non‐medical, health‐related need.

### 3.6 Search methods and sources

We designed a search strategy with an information scientist (DS). We will search the following databases from inception with no date or language restrictions:

- Ovid MEDLINE ALL
- Ovid Embase
- Ovid EBM Reviews—Cochrane Central Register of Controlled Trials
- Elsevier Scopus
- APA PsycINFO
- Clarivate ProQuest (All databases offered by the University of Ottawa, including: ProQuest Dissertations & Theses Global; APA PsycINFO; APA PsycArticles; APA PsycBooks; Canadian Research Index; Coronavirus Research Database; ERIC; International Bibliography of the Social Sciences; Nursing & Allied Health Premium; Publicly Available Content Index; Sociology Collection)
- Epistemonikos
- EBSCO CINAHL
- EBSCO Ageline
- EBSCO platform (All databases offered by the University of Ottawa, including: CINAHL; Academic Search Complete; AgeLine; EconLit; Global Health; Humanities & Social Sciences Index Retrospective: 1907‐1984 (H.W. Wilson); SPORTDiscus with Full Text)
- CABI CAB Direct
- NIHR PROSPERO
- WHO Global Index Medicus
- Clarivate Web of Science platform (Social Sciences Citation Index, Conference Proceedings Citation Index—Social Sciences & Humanities, Emerging Sources Citation Index, Clarivate Korean Citation Index (KCI), Clarivate SciELO Citation Index)
- Google Scholar via Harzing Publish or Perish

We describe the full search strategies in Supporting Information: Appendix 1.

We will screen reference lists of all included systematic reviews in EPPI‐Reviewer to identify additional studies. We will also consult with stakeholders for information about ongoing studies. We will conduct forward and backward citation searching using the Citationchaser Shiny app tool (Haddaway, 2022). We will use the list of included articles as starting articles to search for additional relevant articles that may have been missed through the electronic database searches.

### 3.7 Analysis and presentation

#### 3.7.1 Report structure

Our report will adhere to the following standard sections: abstract, plain language summary, background, methods, results, discussion, and conclusions. We will include in the report a flow chart of included and excluded studies, and a list of on‐going studies waiting for assessment.

We will present this information in the PRISMA 2.0 flow chart and include tables and figures to summarize the distribution of primary studies and systematic reviews, types of interventions and populations identified, outcomes, settings, and geographic distribution.

We will present the evidence gap map with interventions as the rows and the outcomes as the column. The studies will be presented as bubbles with sizes corresponding to the number of included studies and colours to differentiate between primary studies and reviews while considering the methodological quality. We will select the filters in the map based on the number of included studies and consultation with the advisory board.

#### 3.7.2 Filters for presentation

We will use the following as filters in the map:

- Study characteristics: year of publication, publication status of the included study, study design, methodological quality of systematic reviews, reviews with no relevant studies, WHO regions (Africa, Regions of the Americas, South‐East Region, European Region, Eastern Mediterranean Region, Western Pacific Region), Income classification according to the World Bank (low‐income economies, lower‐middle income economies, upper‐middle income economies, high‐income economies), and community setting (e.g., community centre, park, residential or personal home, long‐term care or nursing home and assisted living facilities).
- Intervention characteristics: model characteristics including type of connector (health professional, link worker), point of connection (hospital, home, community), codesign element or empowerment, technology‐based, social prescribing or social prescription.
- Population characteristics: sociodemographics using PROGRESS‐Plus factors and their non‐medical, health‐related social needs.

*Equity analysis*

We will describe population sociodemographics using the PROGRESS‐Plus factors.

We will capture studies that are focused on populations that are at risk of experiencing barriers to health and social care or health inequities. We will use PROGRESS‐Plus acronym to describe factors associated with health inequities (O'Neill, 2014). For these studies, we will document how potential vulnerable older people are defined and identified (e.g., using case finding, outreach, screening). Similarly, we will code for analyses that aim to understand potential differences across any PROGRESS‐Plus factors.

#### 3.7.3 Dependency

Multiple reports of the same study will be treated as one study. A study consisting of multiple interventions or outcomes will be displayed accordingly on the map for each intervention or outcome identified. We will code all relevant categories for interventions with multiple components that fall into multiple categories. We will map systematic reviews to the interventions and outcomes as defined by the question of the systematic review.

### 3.8 Data collection and analysis

#### 3.8.1 Screening and study selection

Two reviewers will independently screen titles and abstracts and full text of potentially eligible articles using EPPI‐Reviewer software and disagreements will be resolved by discussion. We will screen systematic reviews based on their population, intervention, comparison, outcomes (PICO) framework.

We will use machine learning text mining function in EPPI‐Reviewer software to assist in screening at the title and abstract stage. We will deploy a priority screening function that develops a classifier based on the probability of inclusion determined by manually screening approximately 10% of the titles and abstracts. All the eligible studies will be screened independently by two reviewers. We will also screen the reference list of eligible systematic reviews to identify additional studies.

#### 3.8.2 Data extraction and management

We will develop and pilot test a data collection form in EPPI‐Reviewer (Draft in Supporting Information: Appendix 2). After the pilot test, members of the team will individually extract and code the data and a sample of the data extraction and coding will be validated by a second reviewer. Data extraction for systematic reviews will be based on eligible included studies. We will not use any automatic function for coding. The coding categories include study characteristics (study design, publication status, methodological quality assessment of systematic reviews), population description, intervention categories (lifestyle support, psychosocial support, material support, arts‐based activities, nature, others) and subcategories, outcome domains and subdomains, setting and location (countries, World Health Organization regions and World Bank classification by income).

We will aim to capture information describing the population using the PROGRESS‐Plus framework. This acronym stands for Place of residence (urban/rural), Race/ethnicity/culture and language, Occupation, Gender or sex, Religion, Occupation, Socioeconomic status, Social capital (marital status) and additional (plus) factors such as age, health condition, frailty, disabilities and living situation.

We will not attempt to contact organizations or authors of studies and systematic reviews for missing information given the expected size of the map. We will code the available information and discuss scarcity of information, especially for on‐going studies in the report.

#### 3.8.3 Tools for assessing risk of bias/study quality of included reviews

We will assess the methodological quality of systematic reviews using the AMSTAR 2 tool (Shea, 2017) in duplicate. Disagreements will be resolved by discussion. We will not assess the risk of bias of primary studies due to the expected volume of studies. However, following the guidance for evidence and gap maps (Snilstveit, 2016; White, 2020), we will code the study designs as RCTs and non‐randomized studies.

#### 3.8.4 Methods for mapping

The evidence and gap map will be developed using the EPPI‐Mapping tool (Digital Solution Foundry and EPPI_Centre 2020).

## CONTRIBUTIONS OF AUTHORS

- Content: VW, EG, MY, SB, KC, KM, MN, MS, CM, SH, CY, PH.
- EGM methods: VB, MY, OD, VW, EG, PH.
- Information retrieval: DS.

## DECLARATIONS OF INTEREST

VW is Editor in chief of Campbell Collaboration and declares that she will have no role in the editorial process for this manuscript or the decision to publish.

SH is affiliated with the Canadian Institute for Social Prescribing which is anchored by the Canadian Red Cross with funding from the Public Health Agency of Canada and to support collaboration, practices, research and policy that advance integrated health and social care for population wellbeing.

KM is affiliated with the Canadian Institute for Social Prescribing and the Canadian Red Cross.

PH is employed as an advisor to the Canadian Red Cross which provides social prescribing programs to communities across Canada.

EG, MY, OD, VB, SB, KC, CM, MN, MS, DS, CY have no conflicts of interests.

## PLANS FOR UPDATING THE EGM

The EGM will be updated every 2 years.

## SOURCES OF SUPPORT

### Internal sources

- None, Other

### External sources

- Public Health Agency of Canada, Canada

## Supporting information

Supporting information.
