# Supplementary material for: PROTOCOL: Effects of social prescribing for older adults: An evidence and gap map
Source: Campbell Syst Rev. 2024 Feb 29;20(2):e1382. doi: 10.1002/cl2.1382 (PMC10903187; doi:10.1002/cl2.1382)
Supplement: Supplementary file 1 — Supporting information. [file CL2-20-e1382-s001.docx]

# Appendices

## Appendix 1. Search Strategy

Database: Ovid MEDLINE(R) ALL <1946 to November 04, 2022>

Search Date: 5 November 2022

--------------------------------------------------------------------------------

1 community support/ (61)

2 psychosocial support systems/ (929)

3 social prescri*.mp. (363)

4 prescrib* social.tw,kf. (22)

5 (communit* adj2 connect*).tw,kf. (1388)

6 (communit* adj2 referral*).tw,kf. (915)

7 ((community adj2 support) or (community adj2 supports)).tw,kf. (5760)

8 ((health or social) adj2 bridg*).tw,kf. (681)

9 link worker*.tw,kf. (117)

10 (navigat* adj5 social).tw,kf. (930)

11 ((non-clinical or non-medical) adj5 (community or intervention* or provider* or refer* or service* or staff)).tw,kf. (1640)

12 or/1-11 (12474)

13 exp aged/ or middle aged/ (5512294)

14 exp geriatrics/ (31264)

15 (advanced years or ageing or aging or care home* or community-dwelling or elders or elderly or frail* or geriatric* or "late life" or "later life" or "mature adult*" or "middle age*" or "mid* life" or midlife or nursing care or nursing home* or old age* or "oldest old" or patient age* or pensioner* or postmenopaus* or post-menopaus* or retired or retiree* or senior citizen*).ti,kf. (417505)

16 (advanced years or ageing or aging or care home* or community-dwelling or elders or elderly or frail* or geriatric* or "late life" or "later life" or "mature adult*" or "middle age*" or "mid* life" or midlife or nursing care or nursing home* or old age* or "oldest old" or patient age* or pensioner* or postmenopaus* or post-menopaus* or retired or retiree* or senior citizen*).ab. /freq=2 (307129)

17 (older adj3 (adult? or client* or female? or individual* or inpatient* or male? or men or outpatient* or patient* or people or person* or population* or resident* or women)).tw,kf. (291856)

18 ("60 year*" or "over 60" or "65 year*" or "over 65" or "over 70" or "over 75" or "over 80" or "over 85" or "85 year*").tw,kf. (248115)

19 (sexagenarian* or septuagenarian* or octogenarian* or nonagenarian* or centenarian* or supercentenarian*).tw,kf. (7680)

20 (aging or ageing or elder* or geriatric* or gerontolog*).jw,nw. (196666)

21 or/13-20 (5881574)

22 systematic review.mp,pt. (277318)

23 meta analysis.mp,pt. (258201)

24 (cochrane or embase or medline or pubmed or searched).ab. (365065)

25 randomized controlled trial.pt. (579949)

26 controlled clinical trial.pt. (95083)

27 pragmatic clinical trial.pt. (2153)

28 randomi*.tw,kf. (772035)

29 placebo.ab. (232922)

30 clinical trials as topic/ (200534)

31 (randomly adj2 (allocated or assigned)).ab. (164671)

32 trial.ti. (273031)

33 (group or groups).ab. (4167674)

34 ((quasi experiment* or quasiexperiment* or quasi randomi* or quasirandomi*) adj2 (design* or method* or study or trial)).ab,kf. (13888)

35 ((before adj5 after) or (controlled adj3 study) or (controlled adj3 trial) or control group* or effect? or evaluat* or experience* or impact? or intervention* or (pre adj5 post) or ((pretest or pre-test) and (posttest or post test))).ab,kf. /freq=2 (5205362)

36 controlled before-after studies/ (705)

37 (controlled before* adj2 after).ab,kf. (1227)

38 or/22-37 (8620657)

39 animals/ not (humans/ and animals/) (5027074)

40 38 not 39 (7235141)

41 12 and 21 and 40 (2174)

--------------------------------------------------------------------------------

Database: Embase Classic+Embase <1947 to 2022 November 03>

Search Date: 5 November 2022

--------------------------------------------------------------------------------

1 community support/ (331)

2 *psychosocial care/ (4443)

3 social prescri*.mp. (441)

4 prescrib* social.tw,kf. (25)

5 (communit* adj2 connect*).tw,kf. (1670)

6 (communit* adj2 referral*).tw,kf. (1507)

7 ((community adj2 support) or (community adj2 supports)).tw,kf. (7615)

8 ((health or social) adj2 bridg*).tw,kf. (700)

9 link worker*.tw,kf. (151)

10 (navigat* adj5 social).tw,kf. (1201)

11 ((non-clinical or non-medical) adj5 (community or intervention* or provider* or refer* or service* or staff)).tw,kf. (2539)

12 or/1-11 (20004)

13 exp aged/ or middle aged/ (4901243)

14 exp *geriatrics/ (33085)

15 elderly.hw. (323058)

16 (advanced years or ageing or aging or care home* or community-dwelling or elders or elderly or frail* or geriatric* or "late life" or "later life" or "mature adult*" or "middle age*" or "mid* life" or midlife or nursing care or nursing home* or old age* or "oldest old" or patient age* or pensioner* or postmenopaus* or post-menopaus* or retired or retiree* or senior citizen*).ti,kf. (557987)

17 (advanced years or ageing or aging or care home* or community-dwelling or elders or elderly or frail* or geriatric* or "late life" or "later life" or "mature adult*" or "middle age*" or "mid* life" or midlife or nursing care or nursing home* or old age* or "oldest old" or patient age* or pensioner* or postmenopaus* or post-menopaus* or retired or retiree* or senior citizen*).ab. /freq=2 (434773)

18 (older adj3 (adult? or client* or female? or individual* or inpatient* or male? or men or outpatient* or patient* or people or person* or population* or resident* or women)).tw,kf. (404720)

19 ("60 year*" or "over 60" or "65 year*" or "over 65" or "over 70" or "over 75" or "over 80" or "over 85" or "85 year*").tw,kf. (394479)

20 (sexagenarian* or septuagenarian* or octogenarian* or nonagenarian* or centenarian* or supercentenarian*).tw,kf. (11440)

21 (aging or ageing or elder* or geriatric* or gerontolog*).jw. (247040)

22 or/13-21 (5512986)

23 systematic review.tw,kf. (297953)

24 meta analysis.tw,kf. (279622)

25 (cochrane or embase or medline or pubmed or searched).ab. (467060)

26 randomized controlled trial/ (737780)

27 crossover procedure/ (72324)

28 double-blind procedure/ (202921)

29 randomi*.tw,kf. (1110357)

30 placebo.ab. (344370)

31 randomly.ab. (525375)

32 (singl* blind* or doubl* blind*).tw,kf. (268851)

33 ((quasi experiment* or quasiexperiment* or quasi randomi* or quasirandomi*) adj2 (design* or method* or study or trial)).ab,kf. (16911)

34 ((before adj5 after) or (controlled adj3 study) or (controlled adj3 trial) or control group* or effect? or evaluat* or experience* or impact? or intervention* or (pre adj5 post) or ((pretest or pre-test) and (posttest or post test))).ab,kf. /freq=2 (7250336)

35 trial.tw. (1063267)

36 groups.ab. (3481526)

37 (control* adj2 (design or studies or study)).tw,kf. (382864)

38 (controlled before* adj2 after).ab,kf. (1401)

39 or/23-38 (10667498)

40 (exp animal/ or animal.hw. or nonhuman/) not (exp human/ or human cell/ or (human or humans).ti.) (7738879)

41 39 not 40 (8728743)

42 12 and 22 and 41 (2596)

--------------------------------------------------------------------------------

Database: EBM Reviews - Cochrane Central Register of Controlled Trials <September 2022>

Search Date: 5 November 2022

--------------------------------------------------------------------------------

1 social prescri*.mp. (25)

2 prescrib* social.mp. (2)

3 (communit* adj2 connect*).mp. (136)

4 (communit* adj2 referral*).mp. (440)

5 ((community adj2 support) or (community adj2 supports)).mp. (593)

6 ((health or social) adj2 bridg*).mp. (37)

7 link worker*.mp. (19)

8 (navigat* adj5 social).mp. (106)

9 ((non-clinical or non-medical) adj5 (community or intervention* or provider* or refer* or service* or staff)).mp. (195)

10 or/1-9 (1504)

11 (advanced years or ageing or aging or care home* or community-dwelling or elders or elderly or frail* or geriatric* or "late life" or "later life" or "mature adult*" or "middle age*" or "mid* life" or midlife or nursing care or nursing home* or old age* or "oldest old" or patient age* or pensioner* or postmenopaus* or post-menopaus* or retired or retiree* or senior citizen*).mp. (474618)

12 (older adj3 (adult? or client* or female? or individual* or inpatient* or male? or men or outpatient* or patient* or people or person* or population* or resident* or women)).mp. (38931)

13 ("60 year*" or "over 60" or "65 year*" or "over 65" or "over 70" or "over 75" or "over 80" or "over 85" or "85 year*").tw,kf. (68682)

14 (sexagenarian* or septuagenarian* or octogenarian* or nonagenarian* or centenarian* or supercentenarian*).mp. (217)

15 or/11-14 (520642)

16 10 and 15 (478)

--------------------------------------------------------------------------------

Database: Elsevier Scopus

Search Date: 5 November 2022

Yield: 266

--------------------------------------------------------------------------------

( ALL ( ( "social prescri*" OR "prescri* social*" OR "communit* connect*" OR "communit* referr*" OR "link worker*" OR "non-clinical referr*" OR "non-medical referr*" ) ) AND TITLE-ABS-KEY ( ( "advanced years" OR ageing OR aging OR "care home" OR "community-dwelling" OR elders OR elderly OR frail* OR geriatric* OR "late life" OR "later life" OR "mature adult*" OR "middle age*" OR "mid* life" OR midlife OR "nursing care" OR "nursing home*" OR "old age*" OR "oldest old" OR pensioner* OR postmenopaus* OR "post-menopaus*" OR retired OR retiree* OR "senior citizen*" ) ) AND TITLE-ABS-KEY ( ( "systematic review*" OR "meta-analysis" OR "metaanalysis" OR randomi* OR randomly OR double-blind* OR single-blind* OR placebo* OR quasi-experimental OR quasi-randomi* OR "controlled study" OR "controlled trial" OR "control group" OR "pre-test" OR "post-test" ) ) )

--------------------------------------------------------------------------------

Database: APA PsycInfo <1806 to November Week 1 2023>
Search Date: 17 November 2023
Yield: 1348
--------------------------------------------------------------------------------
1 *social support/ (32878)
2 social support*.ti. or social support*.ab. /freq=2 (28972)
3 social prescribing/ (75)
4 social prescri*.tw. (173)
5 prescrib* social.tw. (40)
6 (communit* adj2 connect*).tw. (1745)
7 (communit* adj2 referral*).tw. (445)
8 ((community adj2 support) or (community adj2 supports)).tw. (5757)
9 ((health or social) adj2 bridg*).tw. (904)
10 link worker*.tw. (58)
11 men's shed?.tw. (56)
12 (navigat* adj5 social).tw. (1643)
13 ((non-clinical or non-medical) adj5 (community or intervention* or provider* or refer* or service* or staff)).tw. (709)
14 or/1-13 (54193)
15 older adulthood/ (16513)
16 (advanced years or ageing or aging or care home* or community-dwelling or elders or elderly or frail* or geriatric* or "late life" or "later life" or "mature adult*" or "middle age*" or "mid* life" or midlife or nursing care or nursing home* or old age* or "oldest old" or patient age* or pensioner* or postmenopaus* or post-menopaus* or retired or retiree* or senior citizen*).ti. (87006)
17 (advanced years or ageing or aging or care home* or community-dwelling or elders or elderly or frail* or geriatric* or "late life" or "later life" or "mature adult*" or "middle age*" or "mid* life" or midlife or nursing care or nursing home* or old age* or "oldest old" or patient age* or pensioner* or postmenopaus* or post-menopaus* or retired or retiree* or senior citizen*).ab. /freq=2 (90658)
18 (older adj3 (adult? or client* or female? or individual* or inpatient* or male? or men or outpatient* or patient* or people or person* or population* or resident* or women)).tw. (113788)
19 ("60 year*" or "over 60" or "65 year*" or "over 65" or "over 70" or "over 75" or "over 80" or "over 85" or "85 year*").tw. (33114)
20 (sexagenarian* or septuagenarian* or octogenarian* or nonagenarian* or centenarian* or supercentenarian*).tw. (845)
21 (aging or ageing or elder* or geriatric* or gerontolog*).jw. (86299)
22 or/15-21 (247734)
23 "systematic review"/ or (systematic adj2 review).tw. (49932)
24 meta analysis/ or (meta-analys* or metaanalys*).tw. (50311)
25 (cochrane or embase or medline or pubmed or searched).ab. (48865)
26 exp randomized controlled trials/ (1522)
27 randomi*.tw. (111282)
28 placebo.ab. (43503)
29 (randomly adj2 (allocated or assigned)).ab. (48334)
30 trial.ti. (38779)
31 (group or groups).ab. (995607)
32 ((quasi experiment* or quasiexperiment* or quasi randomi* or quasirandomi*) adj2 (design* or method* or study or trial)).ab. (10838)
33 ((before adj5 after) or (controlled adj3 study) or (controlled adj3 trial) or control group* or effect? or evaluat* or experience* or impact? or intervention* or (pre adj5 post) or ((pretest or pre-test) and (posttest or post test))).ab. /freq=2 (1347740)
34 (controlled before* adj2 after).ab. (132)
35 or/23-34 (2075946)
36 14 and 22 and 35 (2961)
37 or/15-20 (218961)
38 14 and 35 and 37 (2686)
39 limit 38 to "remove medline records" (1348)

--------------------------------------------------------------------------------

Database: Clarivate ProQuest (all databases, including PsycINFO, PsycArticles, IBSS, Dissertations & Theses...)

Search Date: 5 November 2022

Yield: 165

--------------------------------------------------------------------------------

("social prescri*" OR "prescri* social*" OR "communit* connect*" OR "communit* referr*" OR "link worker*" OR "non-clinical referr*" OR "non-medical referr*") AND noft(("advanced years" OR ageing OR aging OR "care home*" OR "community-dwelling" OR elders OR elderly OR frail* OR geriatric* OR "late life" OR "later life" OR "mature adult*" OR "middle age*" OR "mid* life" OR midlife OR "nursing care" OR "nursing home*" OR "old age*" OR "oldest old" OR pensioner* OR postmenopaus* OR "post-menopaus*" OR retired OR retiree* OR "senior citizen*")) AND noft(("systematic review*" OR "meta-analysis" OR "metaanalysis" OR randomi* OR randomly OR double-blind* OR single-blind* OR placebo* OR quasi-experimental OR quasi-randomi* OR "controlled study" OR "controlled trial" OR "control group" OR "pre-test" OR "post-test"))

--------------------------------------------------------------------------------

Database: Epistemonikos

Search Date: 5 November 2022

Yield: 131

-------------------------------------------------------------------

(title:(("social prescribing" OR "social prescription" OR "social prescriptions" OR "prescribing social" OR "communitity connector" OR "community connectors" OR "community referral" OR "community referrals" OR "link worker" OR "link workers" OR "non-clinical referral" OR "non-clinical referrals" OR "non-medical referral" OR "non-medical referrals" OR "prescription social")) OR abstract:(("social prescribing" OR "social prescription" OR "social prescriptions" OR "prescribing social" OR "communitity connector" OR "community connectors" OR "community referral" OR "community referrals" OR "link worker" OR "link workers" OR "non-clinical referral" OR "non-clinical referrals" OR "non-medical referral" OR "non-medical referrals" OR "prescription social")))

--------------------------------------------------------------------------------

Database: EBSCO CINAHL
Search Date: 17 November 2023
Yield: 1268
--------------------------------------------------------------------------------
S21 S20 Limiters - Exclude MEDLINE records 1,268
S20 S4 AND S7 AND S19 2,464
S19 (S8 OR S9 OR S10 OR S11 OR S12 OR S13 OR S14 OR S15 OR S16 OR S17 OR S18) 1,604,173
S18 TI (controlled before* N2 after) OR AB (controlled before* N2 after) 969
S17 AB ((before N3 after) OR (controlled N3 study) OR (controlled N3 trial) OR control group* OR intervention* OR (pre N3 post) OR ((pretest OR pre-test) and (posttest OR post test))) 797,309
S16 MH ("Controlled Before-After Studies") 235
S15 TI ( ((quasi experimental OR quasiexperimental OR quasi randomi* OR quasirandomi*) N2 (design* OR method* OR study OR trial)) ) OR AB ( ((quasi experimental OR quasiexperimental OR quasi randomi* OR quasirandomi*) N2 (design* OR method* OR study OR trial)) ) 15,915
S14 TI ( ((singl* N1 blind*) OR (doubl* N1 blind*)) ) OR AB ( ((singl* N1 blind*) OR (doubl* N1 blind*)) ) 58,182
S13 AB (allocat* OR assign* OR control* OR random*) 981,206
S12 (MH "Randomized Controlled Trials") OR (MH "Single-Blind Studies")OR (MH "Double-Blind Studies") OR (MH "Triple-Blind Studies") OR (MH "Random Assignment") OR MH ("Intervention Trials") OR MH ("Crossover Design") OR MH ("Cluster Sample") OR MH ("Placebos") 233,034
S11 PT randomized controlled trial 153,537
S10 AB (cochrane OR embase OR medline OR pubmed OR searched) 144,768
S9 TI ( (systematic N2 review) OR TI meta analy* OR TI metaanaly* ) OR AB ( (systematic N2 review) OR TI meta analy* OR TI metaanaly* ) 186,411
S8 (MH "Systematic Review") OR (MH "Meta Analysis") 158,749
S7 S5 OR S6 1,022,278
S6 TI ("advanced years" OR ageing OR aging OR "care home*" OR "community-dwelling" OR elders OR elderly OR frail* OR geriatric* OR "late life" OR "later life" OR "mature adult*" OR "middle age*" OR "mid* life" OR midlife OR "nursing care" OR "nursing home*" OR "old age*" OR "oldest old" OR pensioner* OR postmenopaus* OR "post-menopaus*" OR retired OR retiree* OR "senior citizen*") 173,077
S5 (MH "Aged+") 951,339
S4 S1 OR S2 OR S3 37,510
S3 TI ( ("social support*" OR "prescri* social*" OR "communit* connect*" OR "communit* referr*" OR "communit* support*" OR "link worker*" OR "men's shed*" OR "non-clinical referr*”"OR "non-medical referr*") ) OR AB ( ("social support*" OR "prescri* social*" OR "communit* connect*" OR "communit* referr*" OR "communit* support*" OR "link worker*" OR "men's shed*" OR "non-clinical referr*”"OR "non-medical referr*") ) 9,369
S2 TX "social prescribing" 314
S1 (MJ "Support, Psychosocial") 34,290

--------------------------------------------------------------------------------

Database: EBSCO AgeLine
Search Date: 17 November 2023
Yield: 432
--------------------------------------------------------------------------------
S6 S3 AND S4 AND S5 432
S5 TX ("systematic review*" OR "meta-analysis" OR "metaanalysis" OR randomi* OR randomly OR double-blind* OR single-blind* OR placebo* OR quasi-experimental OR quasi-randomi* OR "controlled study" OR "controlled trial" OR "control group" OR "pre-test" OR "post-test") 13,426
S4 TX ("advanced years" OR ageing OR aging OR "care home*" OR "community-dwelling" OR elders OR elderly OR frail* OR geriatric* OR "late life" OR "later life" OR "mature adult*" OR "middle age*" OR "mid* life" OR midlife OR "nursing care" OR "nursing home*" OR "old age*" OR "oldest old" OR pensioner* OR postmenopaus* OR "post-menopaus*" OR retired OR retiree* OR "senior citizen*") 157,553
S3 S1 OR S2 7,566
S2 TX ("social prescri*" OR "social support*" OR "prescri* social*" OR "communit* connect*" OR "communit* referr*" OR "communit* support*" OR "link worker*" OR "men's shed*" OR "non-clinical referr*”"OR "non-medical referr*") 7,566
S1 SU social prescribing 5

--------------------------------------------------------------------------------

Database: EBSCO (all databases, including CINAHL, Academic Search Complete, AgeLine, EconLit, SPORTDiscus...)

Search Date: 7 November 2022

Yield: 451 (376 after removal of duplicates)

--------------------------------------------------------------------------------

S4 S1 AND S2 AND S3 451

S3 ("systematic review*" OR "meta-analysis" OR "metaanalysis" OR randomi* OR randomly OR double-blind* OR single-blind* OR placebo* OR quasi-experimental OR quasi-randomi* OR "controlled study" OR "controlled trial" OR "control group" OR "pre-test" OR "post-test") 2,621,079

S2 ("advanced years" OR ageing OR aging OR "care home*" OR "community-dwelling" OR elders OR elderly OR frail* OR geriatric* OR "late life" OR "later life" OR "mature adult*" OR "middle age*" OR "mid* life" OR midlife OR "nursing care" OR "nursing home*" OR "old age*" OR "oldest old" OR pensioner* OR postmenopaus* OR "post-menopaus*" OR retired OR retiree* OR "senior citizen*") 3,627,969

S1 TX ("social prescri*" OR "prescri* social*" OR "communit* connect*" OR "communit* referr*" OR "communit* support*" OR "link worker*" OR “non-clinical referr*” OR “non-medical referr*”) 164,330

--------------------------------------------------------------------------------

Database: CABI CAB Direct

Search Date: 8 November 2022

Yield: 51

--------------------------------------------------------------------------------

(("social prescri*" OR "prescri* social*" OR "communit* connect*" OR "communit* referr*" OR "communit* support*" OR "link worker*" OR “non-clinical referr*” OR “non-medical referr*”)) AND (("advanced years" OR ageing OR aging OR "care home*" OR "community-dwelling" OR elders OR elderly OR frail* OR geriatric* OR "late life" OR "later life" OR "mature adult*" OR "middle age*" OR "mid* life" OR midlife OR "nursing care" OR "nursing home*" OR "old age*" OR "oldest old" OR pensioner* OR postmenopaus* OR "post-menopaus*" OR retired OR retiree* OR "senior citizen*")) AND (("systematic review*" OR "meta-analysis" OR "metaanalysis" OR randomi* OR randomly OR double-blind* OR single-blind* OR placebo* OR quasi-experimental OR quasi-randomi* OR "controlled study" OR "controlled trial" OR "control group" OR "pre-test" OR "post-test"))

--------------------------------------------------------------------------------

Database: NIHR PROSPERO

Search Date: 8 November 2022

Yield: 41

--------------------------------------------------------------------------------

#1 ("social prescri*" OR "prescri* social*" OR "communit* connect*" OR "communit* referr*" OR "link worker*" OR "non-clinical referr*" OR "non-medical referr*") 124

#2 ("advanced years" OR ageing OR aging OR "care home*" OR "community-dwelling" OR elders OR elderly OR frail* OR geriatric* OR "late life" OR "later life" OR "mature adult*" OR "middle age*" OR "mid* life" OR midlife OR "nursing care" OR "nursing home*" OR "old age*" OR "oldest old" OR pensioner* OR postmenopaus* OR "post-menopaus*" OR retired OR retiree* OR "senior citizen*") 19481

#3 ("systematic review*" OR "meta-analysis" OR "metaanalysis" OR randomi* OR randomly OR double-blind* OR single-blind* OR placebo* OR quasi-experimental OR quasi-randomi* OR "controlled study" OR "controlled trial" OR "control group" OR "pre-test" OR "post-test") 178271

#4 #1 AND #2 AND #3 41

--------------------------------------------------------------------------------

Database: WHO Global Index Medicus

Search Date: 8 November 2022

Yield: 5

--------------------------------------------------------------------------------

(tw:(("social prescribing" OR "social prescription" OR "social prescriptions" OR "prescribing social" OR "communitity connector" OR "community connectors" OR "community referral" OR "community referrals" OR "link worker" OR "link workers" OR "non-clinical referral" OR "non-clinical referrals" OR "non-medical referral" OR "non-medical referrals" OR "prescription social"))) AND (tw:(("advanced years" OR ageing OR aging OR "care home" OR "care homes" OR "community-dwelling" OR elders OR elderly OR frail* OR geriatric* OR "late life" OR "later life" OR "mature adult" OR "mature adults" OR "middle-aged" OR "mid-life" OR midlife OR "nursing care" OR "nursing home" OR "nursing homes" OR "old age" OR "oldest old" OR pensioner* OR postmenopaus* OR "post-menopaus*" OR retired OR retiree* OR "senior citizen" OR "senior citizens")))

--------------------------------------------------------------------------------

Database: Clarivate Web of Science Social Sciences Citation Index (SSCI), Conference Proceedings Citation Index – Social Science & Humanities (CPCI-SSH), Emerging Sources Citation Index (ESCI)

Search Date: 8 November 2022

Yield: 53

--------------------------------------------------------------------------------

("social prescri*" OR "prescri* social*" OR "communit* connect*" OR "communit* referr*" OR "link worker*" OR "non-clinical referr*" OR "non-medical referr*") (Topic) AND ("advanced years" OR ageing OR aging OR "care home*" OR "community-dwelling" OR elders OR elderly OR frail* OR geriatric* OR "late life" OR "later life" OR "mature adult*" OR "middle age*" OR "mid* life" OR midlife OR "nursing care" OR "nursing home*" OR "old age*" OR "oldest old" OR pensioner* OR postmenopaus* OR "post-menopaus*" OR retired OR retiree* OR "senior citizen*") (Topic) AND ("systematic review*" OR "meta-analysis" OR "metaanalysis" OR randomi* OR randomly OR double-blind* OR single-blind* OR placebo* OR quasi-experimental OR quasi-randomi* OR "controlled study" OR "controlled trial" OR "control group" OR "pre-test" OR "post-test") (All Fields)

--------------------------------------------------------------------------------

Database: Clarivate Korean Citation Index (KCI)

Search Date: 8 November 2022

Yield: 3

--------------------------------------------------------------------------------

("social prescri*" OR "prescri* social*" OR "communit* connect*" OR "communit* referr*" OR "link worker*" OR "non-clinical referr*" OR "non-medical referr*") (Topic) AND ("advanced years" OR ageing OR aging OR "care home*" OR "community-dwelling" OR elders OR elderly OR frail* OR geriatric* OR "late life" OR "later life" OR "mature adult*" OR "middle age*" OR "mid* life" OR midlife OR "nursing care" OR "nursing home*" OR "old age*" OR "oldest old" OR pensioner* OR postmenopaus* OR "post-menopaus*" OR retired OR retiree* OR "senior citizen*") (Topic)

--------------------------------------------------------------------------------

Database: Clarivate SciELO Citation Index

Search Date: 8 November 2022

Yield: 7

--------------------------------------------------------------------------------

("social prescri*" OR "prescri* social*" OR "communit* connect*" OR "communit* referr*" OR "link worker*" OR "non-clinical referr*" OR "non-medical referr*") (Topic) AND ("advanced years" OR ageing OR aging OR "care home*" OR "community-dwelling" OR elders OR elderly OR frail* OR geriatric* OR "late life" OR "later life" OR "mature adult*" OR "middle age*" OR "mid* life" OR midlife OR "nursing care" OR "nursing home*" OR "old age*" OR "oldest old" OR pensioner* OR postmenopaus* OR "post-menopaus*" OR retired OR retiree* OR "senior citizen*") (Topic)

--------------------------------------------------------------------------------

Database: Google Scholar via Harzing Publish or Perish

Search Date: 8 November 2022

Yield: 294

--------------------------------------------------------------------------------

Title: "social prescribing" OR "social prescription" OR "social prescriptions" OR "link worker" OR "link workers"

Keywords: "systematic review" OR "randomized" OR "controlled trial" OR placebo OR "double-blind" OR "single-blind"

--------------------------------------------------------------------------------

Database: EBM Reviews - Cochrane Database of Systematic Reviews <2005 to November 2, 2022>

Search Date: 5 November 2022

--------------------------------------------------------------------------------

1 social prescri*.af. (0)

2 prescrib* social.af. (0)

3 (communit* adj2 connect*).af. (9)

4 (communit* adj2 referral*).af. (21)

5 ((community adj2 support) or (community adj2 supports)).ti,ab,kw. (5)

6 ((health or social) adj2 bridg*).ti,ab,kw. (0)

7 link worker*.af. (7)

8 (navigat* adj5 social).ti,ab,kw. (0)

9 ((non-clinical or non-medical) adj5 (community or intervention* or provider* or refer* or service* or staff)).ti,ab,kw. (7)

10 or/1-9 (48)

11 (advanced years or ageing or aging or care home* or community-dwelling or elders or elderly or frail* or geriatric* or "late life" or "later life" or "mature adult*" or "middle age*" or "mid* life" or midlife or nursing care or nursing home* or old age* or "oldest old" or patient age* or pensioner* or postmenopaus* or post-menopaus* or retired or retiree* or senior citizen*).ti,ab,kw. (1002)

12 (older adj3 (adult? or client* or female? or individual* or inpatient* or male? or men or outpatient* or patient* or people or person* or population* or resident* or women)).ti,ab,kw. (355)

13 ("60 year*" or "over 60" or "65 year*" or "over 65" or "over 70" or "over 75" or "over 80" or "over 85" or "85 year*").ti,ab,kw. (173)

14 (sexagenarian* or septuagenarian* or octogenarian* or nonagenarian* or centenarian* or supercentenarian*).ti,ab,kw. (0)

15 or/11-14 (1273)

16 10 and 15 (11)

## Appendix 2. Coding tool

| Categories | Sub-categories (if applicable) |
| --- | --- |
| Publication status | · Complete  · On-going (e.g., protocols, trial registrations)  · Conference abstract |
| Study design | · Systematic review  · Primary study |
| Primary study design | · Randomized controlled trials (RCTs)  · Non-randomized studies |
| Equity focus: Is the study population identified by the authors as aimed at/focused on disadvantaged across any PROGRESS-Plus factors | · Place of residence (rural or remote/urban)  · Race/Ethnicity/culture/language  · Occupation  · Gender or Sex  · Religion  · Education  · Socioeconomic status  · Social capital (e.g., marital status – widowed, divorced, separated)  · Plus-factor – social isolated or at risk  · Plus-factor – lonely or at risk  · Plus-factor – age (older adults)  · Plus-factor – disability  · Plus-factor – frailty  · Plus-factor – health status (e.g., dementia, disease severity)  · Plus-factor – living situation (e.g., alone, long term care, away from home) |
| If population is identified as “at risk”, how are they identified? | · Case-finding (methods for detecting people who are at risk for a particular condition e.g., assessment using a questionnaire or scale)  · Outreach (e.g., contacting organizations, groups, specific audiences or the general public to identify people who are at risk)  · From a community-based program (people at risk who participate in a community program e.g., meals on wheels, dance classes, exercise programs, book clubs, community garden, community network, etc.)  · Screening in primary care  · Through formal service network or agencies (e.g., Army, Veterans affairs, penitentiary/prisons, employment agencies, institutional networks, schools) |
| Quality assessment of reviews (using AMSTAR 2) | · Critically low-quality reviews  · Low quality reviews  · Moderate quality reviews  · High quality reviews  · Randomized control trials  · Non-randomized studies |
| Interventions | |
| Lifestyle support | · Exercise on prescription  · Counselling on alcohol and drugs |
| Psychosocial support | · Social and emotional support  · Mental health support  · Volunteering  · Intergenerational activities  · Lifelong learning (education and skills building)  · Book reading (bibliotherapy)  · Solidarity networks & social support groups  · Spiritual care |
| Material support | · Housing  · Nutrition and food security  · Transportation  · Financial support  · Legal and citizen advice and support  · Employment services, vocational learning and social entrepreneurship support |
| Arts based activities | · Music  · Museums, galleries, arts & crafts  · Dance |
| Nature | · Gardening (horticulture)  · Local parks prescription  · Green gym/ Deprescribing for greenhouse gas reductions |
| Other | . Population specific or culturally safe programs (e.g., Men’s shed, Black-focused social prescribing program)  · Signposting  · Care navigation programs |
| Intervention characteristics | |
| Type of connector | · Healthcare professional  · Lay person (any trusted individual in the community)  · Researcher  · Unspecified |
| Point of connection | · Medical facility (hospital, clinic, medical centre)  · Home (personal home, independent living, residential home, assisted living, long-term care/nursing home)  · Community (community centre, park, art gallery, museum, workplace)  · Unspecified |
| Codesign element | · Specified  · Unspecified  · No co-design element |
| Empowerment | · Specified  · Unspecified |
| Intervention type | · Social prescribing  · Social prescription |
| Intervention format | · Technology-based  · Non-technology-based  · Unspecified |
| Outcomes | |
| Process outcomes | · Acceptance  · Uptake  · Adherence  · Feasibility |
| Individual outcomes | · Wellbeing (physical, mental, social, general)/quality of life  · Social connections (loneliness, social isolation)  · Anxiety/depression  · Social support  · Social participation  · Physical activity,  · Self-esteem or self-efficacy/ self-management  · Empowerment  · Trust  · Physiological outcomes (e.g., blood glucose levels, cholesterol, BMI, blood pressure) |
| Health systems | · Health/social service use  · Healthcare or social care utilization costs  · Cost-effectiveness  · Health system integration  · Care team wellbeing/experience |
| Community outcomes | · Community resilience  · Community connectedness  · Civic participation  · Social cohesion  · Health and social equity |
| Adverse effects | · Stigma  · Unintended outcomes |
| Population sociodemographic | |
| Age groups | · Includes 60-75 years (youngest-old)  · Includes 75-85 years (middle-old)  · Includes >85 years (oldest-old)  · Restricted to >80 years old  · Unspecified |
| Health condition | · Communicable disease (e.g., respiratory infections, pneumonia)  · Non-communicable disease (e.g., diabetes, hypertension, COPD)  · Dementia  · Depression  · Other mental health disorders  · Comorbidity (multiple health conditions)  · Disability  · Frailty  · Care dependent  · Discharge from hospital  · End of life/palliative care |
| Place of residence | · Urban  · Rural/remote  · Unspecified |
| Race/Ethnicity/Language/Culture | · Tick if reported |
| Occupation | · Tick if reported |
| Gender or Sex | · Men only  · Women only  · LGBTQIA2S+  · Unspecified |
| Religion | · Tick if reported |
| Education | · Tick if reported |
| Socioeconomic status | · Tick if reported |
| Marital status | · Tick if reported |
| Living alone | · Tick if reported |
| Caregivers (target population includes caregivers) | · Tick if reported |
| Non-clinical needs | · Social and emotional needs (social connections)  · Purpose in life (civic engagement, meaningfulness, and status, being able to contribute to society)  · Mobility  · Personal care needs  · Meals  · Domestic assistance  · Accommodation (housing/home modifications and maintenance)  · Financial management  · Communication (language support/interpreters, information and assistance/referral services)  · Lifelong learning and skills development  · Care navigation support or task orientation  · Respite care  · Caregiver support |
| WHO regions | · African Region  · The Americas  · South-East Asia Region  · European Region  · Eastern Mediterranean Region  · Western Pacific Region  · Multiple  · Unspecified (any country is eligible) |
| World Bank income classification | · Low-income economies  · Lower-middle income economies  · Upper-middle income economies  · High-income economies  · Unspecified (any country is eligible) |
| Countries | · Africa (where multiple countries)  · Europe (where multiple countries)  · Afghanistan  · Angola  · Armenia  · Argentina  · Australia  · Austria  · Bahamas, The  · Bahrain  · Bangladesh  · Barbados  · Belgium  · Belarus  · Belize  · Bolivia  · Brazil  · Botswana  · Bulgaria  · Burkina Faso  · Cambodia  · Cameroon  · Canada  · Chile  · China  · Congo  · Croatia  · Cuba  · Colombia  · Cyprus  · Czech Republic  · Dominican Republic  · Denmark  · Egypt  · Ecuador  · Eritrea  · Estonia  · Finland  · Ethiopia  · Gambia, The  · France  · Georgia  · Ghana  · Germany  · Greece  · Guatemala  · Guinea-Bissau  · Haiti  · Honduras  · Hong Kong  · Hungary  · Iceland  · India  · Indonesia  · Iran  · Ireland  · Israel  · Italy  · Ivory Coast  · Jamaica  · Japan  · Jordan  · Kazakhstan  · Kenya  · Korea  · Kuwait  · Lao  · Latin America (where multiple countries)  · Lebanon  · Lesotho  · Liberia  · Latvia  · Lithuania  · Luxembourg  · Macedonia  · Malawi  · Madagascar  · Malaysia  · Mali  · Mexico  · Micronesia  · Marshall Islands  · Mozambique  · Mongolia  · Myanmar (Burma)  · Namibia  · Nepal  · New Zealand  · The Netherlands  · Nicaragua  · Niger  · Nigeria  · Northern Ireland  · Norway  · Pakistan  · Panama  · Papua New Guinea  · Peru  · Philippines  · Poland  · Portugal  · Puerto Rico  · Romania  · Russia  · Rwanda  · Samoa  · San Marino  · Saudi Arabia  · Scotland  · Serbia  · Senegal  · Singapore  · Sierra Leone  · Slovakia  · Slovenia  · South Africa  · Spain  · Sri Lanka  · St Lucia  · Swaziland  · Sweden  · Switzerland  · Syria  · Taiwan  · Thailand  · Tanzania  · Trinidad and Tobago  · Tunisia  · Uganda  · Ukraine  · Turkey  · UK  · USA  · Uzbekistan  · Vanuatu  · Venezuela  · Vietnam  · West Indies  · Yemen  · Zambia  · Zimbabwe |
| Equity analysis | |
| Does the study assess any differences in effects (benefit or harm) across any PROGRESS-Plus factors | · Place of residence (rural or remote/urban)  · Race/Ethnicity/culture/language  · Occupation  · Gender or Sex  · Religion  · Education  · Socioeconomic status  · Social capital (e.g., marital status – widowed, divorced, separated)  · Plus-factor – social isolated or at risk  · Plus-factor – lonely or at risk  · Plus-factor – age (older adults, children)  · Plus-factor – disability  · Plus-factor – frailty  · Plus-factor – health status (e.g., dementia, disease severity)  · Plus-factor – living situation (e.g., alone, long term care, away from home) |
